# Sphingosine‐1‐Phosphate Signalling Inhibition Suppresses Th1‐Like Treg Generation by Reversing Mitochondrial Uncoupling

**DOI:** 10.1111/imm.13870

**Published:** 2024-10-24

**Authors:** Rachel Coulombeau, Claudia Selck, Nicolas Giang, Abdulrahman Al‐Mohammad, Natalie Ng, Allison K. Maher, Rafael Argüello, Antonio Scalfari, James Varley, Richard Nicholas, Margarita Dominguez‐Villar

**Affiliations:** ^1^ Department of Infectious Disease, Faculty of Medicine Imperial College London London UK; ^2^ School of Clinical Medicine, University of Cambridge Cambridge UK; ^3^ Immunometabolism and Translation Aix Marseille Université, CNRS, INSERM, Centre d'Immunologie de Marseille‐Luminy Marseille France; ^4^ Centre of Neuroscience, Department of Medicine Charing Cross Hospital London UK; ^5^ Centre of Neuroscience Imperial College Healthcare NHS Trust London UK

**Keywords:** autoimmunity, EAE/MS, Treg

## Abstract

Inflammatory environments induce the generation of dysfunctional IFNγ^+^T‐bet^+^FOXP3^+^ Th1‐like Tregs, which show defective function and are found in autoimmune conditions including multiple sclerosis (MS). The pathways that control the generation of Th1‐like Tregs are not well understood. Sphingosine‐1‐phosphate (S1P) signalling molecules are upregulated in Th1‐like Tregs, and in vivo S1P inhibition with Fingolimod (FTY720) inhibits the expression of genes responsible for Treg plasticity in MS patients. However, the underlying mechanisms are unknown. Here we show that S1P signalling inhibition by FTY720 inhibits the generation of Th1‐like Tregs and rescues their suppressive function. These effects are mediated by a decrease in mTORC1 signalling and reversal of the mitochondrial uncoupling that Tregs undergo during their reprogramming into Th1‐like Tregs in vitro. Finally, these results are validated in in vivo‐generated Th1‐like Tregs, as Tregs from MS patients treated with FTY720 display decreased Th1‐like Treg frequency, increased suppressive function and mitochondrial metabolism rebalance. These results highlight the involvement of mitochondrial uncoupling in Treg reprogramming and identify S1P signalling inhibition as a target to suppress the generation of dysfunctional Th1‐like Tregs.

## Graphical abstract

Sphingosine‐1‐phosphate signalling blockade inhibits the generation of Th1‐like Tregs via mTORC1 inhibition and reversal of Th1‐like Treg mitochondrial uncoupling.

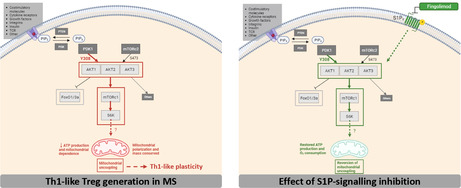

Regulatory T cell (Treg) plasticity occurs as a physiological adaptation to the changing environment and the sensing of danger signals, which allows Tregs to control specific helper T cell responses [1]. However, aberrant Treg acquisition of an effector phenotype with secretion of pro‐inflammatory cytokines is characteristic of several autoimmune conditions including multiple sclerosis (MS) [2, 3], type 1 diabetes [4], rheumatoid arthritis [5], systemic sclerosis [6] and autoimmune hepatitis [7]. MS is an autoimmune disease of the central nervous system (CNS) characterised by infiltration of activated inflammatory cells into the CNS that damage myelin and axons [8]. In MS, Tregs display a Th1‐like phenotype characterised by the increased secretion of the pro‐inflammatory cytokine IFNγ, upregulation of T‐bet and decreased suppressive capacity [3]. The main known pathway involved in the acquisition of a Th1‐like phenotype by Tregs is PI3K/AKT/FOXO signalling [2, 9, 10], but multiple soluble factors, including IL‐12, NaCl and oleic acid [3, 11, 12] and cell surface receptors such as TIGIT and neuropilin‐1 [13, 14] have been shown to either induce or inhibit Treg plasticity towards a Th1‐like phenotype, many of them by feeding into the PI3K/AKT pathway.

Tregs exhibit a unique metabolic profile, relying heavily on lipid metabolism and oxidative phosphorylation for their function [15, 16] and less on glycolysis [17] compared to effector T cells. In support of this, FOXP3 expression has been shown to promote mitochondrial respiration [18], suggesting that proper Treg function requires mitochondrial integrity [19, 20]. For example, mice with a Treg‐specific deletion of complex III of the mitochondrial respiratory chain develop fatal autoimmunity early in life, without affectation of Treg numbers or FOXP3 expression [21].

Sphingosine‐1‐phosphate (S1P) is a bioactive lipid mediator secreted to the extracellular space mostly by vascular endothelial cells, red blood cells and platelets, that is involved in many physiological processes including cell migration, differentiation and survival [22]. S1P signals via five G protein‐coupled receptors, that is S1PR1‐S1PR5, which are expressed on the surface of lymphocytes amongst other cell types [23]. In T cells, S1P signalling mediates the egress of lymphocytes from secondary lymphoid organs to the target tissue via an S1P gradient, where S1P concentrations are low in lymphoid organs but high in blood and lymph [24]. Thus, inhibition of inflammatory T cell migration from the periphery to the CNS by blocking S1P signalling is a successful therapeutic approach in MS [25, 26]. FTY720 (Fingolimod) is a S1P receptor modulator that is phosphorylated in target cells by sphingosine kinases to become biologically active. Once phosphorylated, FTY720 becomes a high affinity ligand for four S1P receptors (all except S1PR2), acting as a functional antagonist and thereby inhibiting lymphocyte egress from secondary lymph nodes [27]. However, increasing number of works suggest that S1P signalling participates in many additional processes besides cell migration [23] including dendritic cell and macrophage function, promotion of T cell differentiation to Th1 and Th17 cells and germinal centre B cell survival [28, 29, 30, 31]. In this respect, we have previously demonstrated that Treg cells isolated from FTY720‐treated relapsing–remitting (RR) MS patients show a decrease in the expression of genes associated with the Th1‐like phenotype ex vivo compared to baseline, suggesting that FTY720 affects Treg stability independently of cell migration [32]. However, the underlying mechanisms are unknown.

Here we investigated the role of S1P signalling on the regulation of dysfunctional Th1‐like Tregs by utilising FTY720 as a pathway inhibitor. We found that Th1‐like Treg generation is suppressed by S1P signalling inhibition by targeting mTORC1 activation. Moreover, Th1‐like Tregs undergo mitochondrial uncoupling, with a decrease in mitochondrial dependence and oxygen consumption rate (OCR) but maintained mitochondrial membrane potential as compared to control Tregs, which is reversed by FTY720 treatment. Finally, by using MS Tregs as a model of in vivo‐generated Th1‐like Tregs, we demonstrate that in vivo FTY720 treatment reverses the mitochondrial dysfunction observed in Tregs from untreated RRMS patients. These results underscore the involvement of mitochondrial uncoupling in Treg plasticity and the role of S1P signalling inhibition in restoring mitochondrial function, suggesting that targeting mitochondrial uncoupling is a potential strategy to control Treg stability.

## 1 Results

### 1.1 S1P Signalling Inhibition Suppresses Th1‐Like Treg Phenotype and Function

**FIGURE 1:**
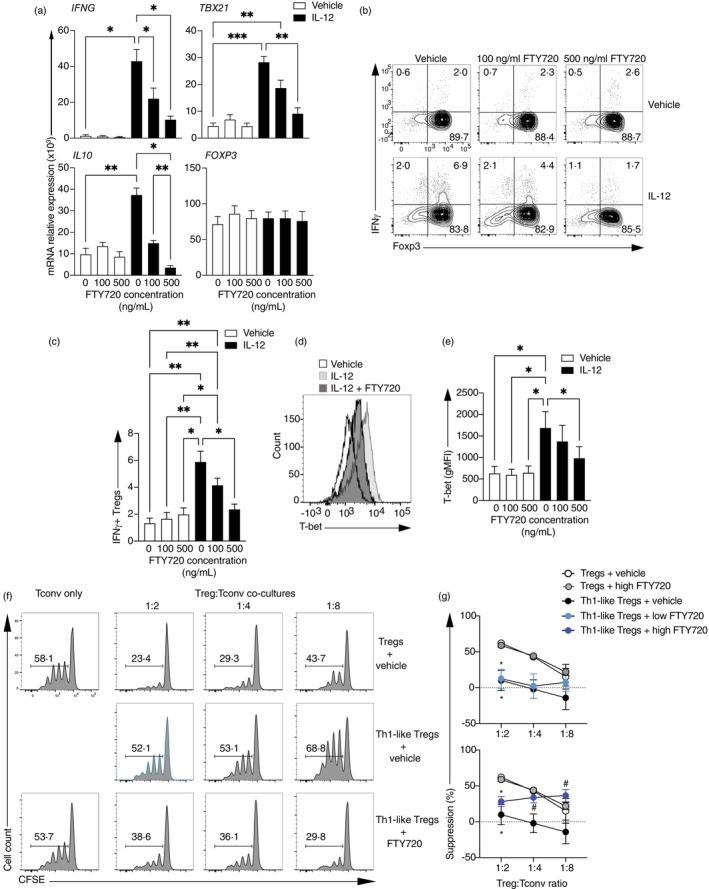
FTY720 inhibits the generation of Th1‐like Tregs in vitro. Sorted Tregs from healthy individuals were stimulated with anti‐CD3, anti‐CD28 and IL‐2 in the presence or absence of IL‐12 and increasing doses of FTY720. (A) Gene expression measured 48 h after activation (*n* = 4–9). Representative example (B) and summary (C) of IFNγ production 4 days after initial activation and a 4 h incubation with PMA and ionomycin in the presence of GolgiStop (*n* = 8). Representative histogram (D) and summary (E) of T‐bet expression measured 48 h after activation and represented as geometric mean fluorescence intensity (gMFI, *n* = 7). (F and G). Sorted Tregs (CD4^+^CD25^high^CD127^low^) were stimulated as above in the presence or absence of IL‐12. Seventy‐two hours later, cells were collected, washed and co‐cultured with Treg‐depleted, CFSE‐labelled CD4^+^ T cells (Tconv) for 3.5 days in the presence of various concentrations of FTY720. (F) Representative histogram of Tconv proliferation in co‐culture at various Treg:Tconv ratios. (G) Summary of percentage of suppression by Tregs and Th1‐like Tregs in the presence or absence of FTY720 at various Treg:Tconv ratios (*n* = 5). One‐way ANOVA or mixed effects model with Tukey's correction for multiple comparisons for (A), (C) and (E). Two‐way ANOVA with Tukeys correction for multiple comparisons in (G). *p < 0.05; **p < 0.01; ***p < 0.005.

S1P signalling inhibition by in vivo FTY720 (Fingolimod) treatment in RRMS patients leads to a decrease in *IFNG* and *TBX21* gene expression [32] and increased expression of TIM‐3, a marker of highly suppressive Tregs [33], by Tregs. As Tregs express S1P receptors [29, 34], we decided to examine whether inhibition of the Th1‐like Treg phenotype observed was due to a direct S1P signalling inhibition on Treg cells. For this, we stimulated Tregs isolated from healthy individuals in the presence or absence of IL‐12, used as a model to induce Th1‐like Tregs in vitro [3, 35], and we tested various concentrations of FTY720 for their effect in regulating Th1‐like Treg markers (Figure 1). FTY720 did not cause any significant increase in cell death as compared to vehicle‐treated Tregs at any of the concentrations used (Figure S1). Whilst no effects of FTY720 were observed in the expression of *IFNG*, *IL10*, *TBX21* or *FOXP3* in vehicle‐treated Tregs, FTY720 significantly decreased the expression of *IFNG*, *TBX21* and *IL10* in Th1‐like Tregs. No changes were observed in the expression of *FOXP3* (Figure 1A). These results were confirmed at protein level, with increasing doses of FTY720 significantly inhibiting the production of IFNγ (Figure 1B,C) whilst not affecting the expression of FOXP3 (Figure 1B and Figure S2). Moreover, IL‐17A and IL‐10 production was not affected by S1P on Th1‐like Tregs and only IL‐10 was significantly increased in control Tregs upon S1P treatment. No effects were observed in proliferation (Figure S3). T‐bet expression was significantly reduced in Th1‐like Tregs treated with FTY720 compared to Th1‐like Tregs, supporting the hypothesis that FTY720 treatment directly inhibits the generation of Th1‐like Tregs (Figure 1D,E). FTY720 can bind S1P receptors 1, 3, 4 and 5 [36], and because S1PR2 and S1PR5 are not expressed on human Tregs (Figure S4), we used specific antagonists of S1PR1 (EX 26), S1PR3 (TY 52156) and S1PR4 (CYM 50358) to further identify the main receptor involved in the inhibition of Th1‐like Treg generation. Using T‐bet expression as a readout for Th1‐like reprogramming, our data suggest that inhibition of both S1PR3 and S1PR4 can mimic the effects observed with FTY720 treatment (Figure S5).

To determine whether the inhibition of Th1‐like Treg phenotype by FTY720 was accompanied by restored Treg function, Th1‐like Tregs were generated in vitro with IL‐12 and further co‐cultured with CFSE‐labelled conventional T cells (Treg‐depleted CD4^+^ T cells, Tconv) in the presence or absence of FTY720. Tconv proliferation by CFSE dilution was measured as a readout for Treg suppressive capacity (Figure 1F, Figure S6). As expected [2, 3], in vitro‐generated Th1‐like Tregs displayed impaired suppressive capacity as compared to Tregs, and this was restored by FTY720 to levels comparable to control Tregs (Figure 1G). These data suggest that inhibition of S1P signalling by FTY720 inhibits the generation and impaired function of Th1‐like Tregs.

### 1.2 S1P Signalling Inhibition Suppresses the Activation of mTORC1

**FIGURE 2:**
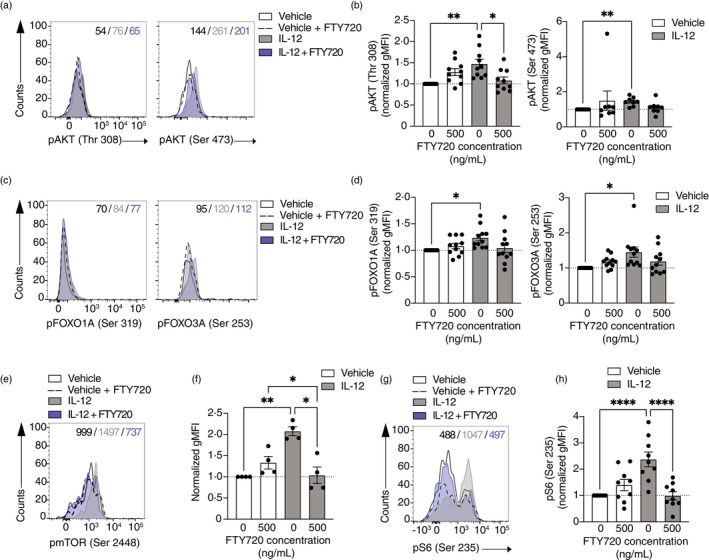
FTY720 inhibits mTORC1 signalling to suppress Th1‐like Treg generation. CD4^+^ T cells from healthy individuals were stimulated with anti‐CD3, anti‐CD28 and IL‐2 in the presence or absence of IL‐12 and increasing doses of FTY720 for 18 h and fixed for cellular staining using FOXP3 and CD25 to identify Tregs. Representative histogram (A) and summary (B) of phosphorylation of AKT measured at Thr 308 (left, *n* = 10) and Ser 473 (right, *n* = 8). Representative histogram (C) and summary (D) of phosphorylation of FOXO1A (left, *n* = 11) and FOXO3A (right, *n* = 11). Representative histogram (E) and summary (F) of phosphorylation of mTOR (*n* = 4). Representative histogram (G) and summary (H) of S6 phosphorylation (*n* = 9). All summary plots are shown as normalised gMFI (to vehicle‐treated Treg values). One‐way ANOVA with Tukey's correction for multiple comparisons for (B), (D), (F) and (H). *p < 0.05; **p < 0.01; ****p < 0.005. gMFI, geometric mean fluorescence intensity.

We went on to examine potential mechanisms underlying the inhibition of Th1‐like Tregs by FTY720. We and others had previously shown that the PI3K/AKT/FOXO pathway is activated in Th1‐like Tregs, and its inhibition restores Treg phenotype and function [2, 9]. Therefore, we examined the activation of AKT as a readout for PI3K signalling in Tregs and Th1‐like Tregs in the presence or absence of FTY720 (Figure 2). Th1‐like Tregs displayed an increased phosphorylation of AKT at Thr 308 and Ser 473, the two residues that are required for full activation of AKT [37] (Figure 2A,B). Interestingly, FTY720 significantly inhibited Thr 308 phosphorylation of AKT, whilst not affecting phosphorylation of Ser 473 (Figure 2B). FOXO1A and FOXO3A are downstream targets of PI3K/AKT and are involved in the generation of Th1‐like Tregs [2, 38]. However, FTY720 did not inhibit the phosphorylation of FOXO1A or FOXO3A in Th1‐like Tregs (Figure 2C,D), suggesting that FTY720 is targeting other AKT downstream targets.

We decided to examine mTORC1 activation because it is downstream of PI3K/AKT signalling and has been implicated in controlling regulatory T cell function [39]. Interestingly, dysfunctional Th1‐like Tregs displayed an increased phosphorylation of mTOR (Figure 2E) that was significantly suppressed with FTY720 in a dose‐dependent manner (Figure 2F). In addition, phosphorylation of S6, a downstream target of mTORC1, was significantly increased in Th1‐like Tregs as compared to Tregs (Figure 2G), and FTY720 significantly decreased its activation to control levels (Figure 2H).

**FIGURE 3:**
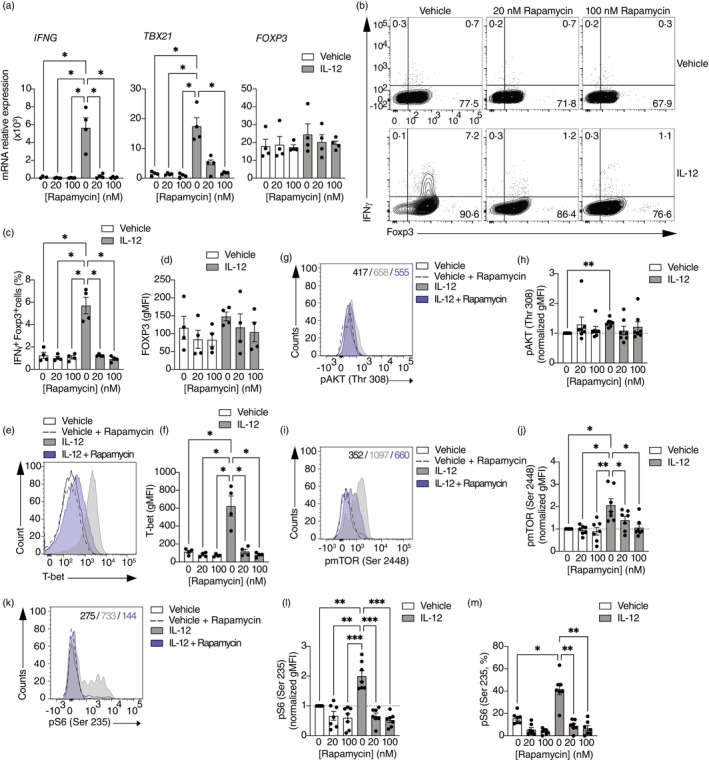
mTORC1 inhibition with rapamycin is sufficient to inhibit Th1‐like Treg reprogramming. Sorted Tregs from healthy individuals were stimulated with anti‐CD3, anti‐CD28 and IL‐2 in the presence or absence of IL‐12 and increasing doses of rapamycin. (A) Gene expression measured 48 h after activation (*n* = 4). Representative example (B) and summary (C) of IFNγ production 4 days after initial activation and a 4 h stimulation with PMA and ionomycin in the presence of GolgiStop (*n* = 4). (D) Summary of FOXP3 expression 48 h after activation (*n* = 4). Representative histogram (E) and summary (F) of T‐bet expression measured 48 h after activation and represented as geometric mean fluorescence intensity (gMFI, *n* = 4). Representative histogram (G) and summary (H) of phosphorylation of AKT measured at Thr 308 (left, *n* = 7). Representative histogram (I) and summary (J) of phosphorylation of mTOR (*n* = 7). Representative histogram (K) and summary of S6 phosphorylation measured as gMFI (L) or frequency of phosphor‐S6+ Tregs (M, *n* = 7). All summary plots are shown as normalised gMFI (to vehicle‐treated Treg values). One‐way ANOVA with Tukey's correction for multiple comparisons for (A), (C), (D), (F), (H), (J) and (L). Only comparisons of Th1‐like Tregs treated with vehicle to all other groups are shown. *p < 0.05; **p < 0.01; ***p < 0.005.

mTORC1 has been shown to act as a negative and positive regulator of Treg development and function, and contrasting works suggest that its functions are associated with a fine tuning of its activity in different contexts [39]. We decided to examine whether inhibition of mTORC1 was sufficient to inhibit Th1‐like Treg generation (Figure 3). For this, we stimulated Tregs and Th1‐like Tregs in the presence of increasing concentrations of rapamycin, an mTORC1 inhibitor. Th1‐like Tregs significantly decreased the expression of *IFNG* and *TBX21* in the presence of rapamycin (Figure 3A), whilst no changes were observed in *FOXP3* gene expression. These results were confirmed at the protein level, with in vitro‐generated Th1‐like Tregs decreasing the production of IFNγ in the presence of rapamycin (Figure 3B,C) whilst maintaining the levels of FOXP3 expression (Figure 3D). In agreement with the decrease in IFNγ production, T‐bet expression was significantly diminished in Th1‐like Tregs in the presence of rapamycin (Figure 3E,F), overall suggesting that mTORC1 inhibition is sufficient to inhibit Th1‐like Treg generation. Mechanistically, rapamycin did not affect the activation of AKT (Thr 308) observed in Th1‐like Tregs (Figure 3G,H), but it significantly decreased the activation of mTOR (Figure 3I,J) and its downstream target S6 (Figure 3K–M).

Overall, these data suggest that FTY720 inhibits Th1‐like Treg generation by suppressing the PI3K/AKT/mTORC1 pathway, and inhibition of mTORC1 by rapamycin is sufficient to suppress IL‐12‐driven Th1‐like Treg generation in vitro.

### 1.3 Th1‐Like Tregs Undergo Mitochondrial Uncoupling That is Reversed by S1P Signalling Inhibition

mTORC1 is an important nutrient sensor that integrates metabolic cues with signalling pathways. It has been shown to control mitochondrial biogenesis and activity, glycolysis and it facilitates the rewiring towards anabolic metabolism required for the activation and expansion of T cells [40]. Mitochondrial function is essential for Treg suppressive capacity [20, 21], and therefore, we decided to explore if mitochondrial metabolism is altered in Th1‐like Tregs, and whether S1P signalling inhibition suppresses Th1‐like Treg generation by modulating it.

**FIGURE 4:**
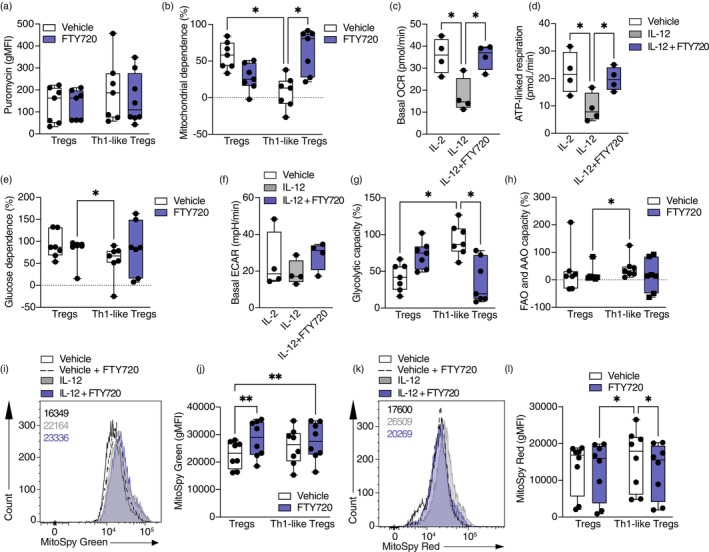
Th1‐like Tregs display mitochondrial uncoupling that is reversed by S1P signalling inhibition. Sorted Tregs from healthy individuals were stimulated with anti‐CD3, anti‐CD28 and IL‐2 in the presence or absence of IL‐12 and FTY720 for 48 h. (A) Summary of puromycin incorporation measured as gMFI (*n* = 7). (B) Summary of percentage of mitochondrial dependence (*n* = 7). (C) Summary of basal oxygen consumption rate (OCR, *n* = 4). (D) Summary of ATP‐linked respiration (*n* = 4). (E) Summary of percentage of glucose dependence (*n* = 7). (F) Summary of basal extracellular acidification rate (*n* = 4). (G) Summary of percentage of glycolytic capacity (*n* = 7). (H) Summary of percentage of fatty acid and amino acid oxidation (*n* = 7). Representative histogram (I) and summary (J) of mitochondrial mass measured by MitoSpy Green incorporation (gMFI, *n* = 8). Representative histogram (K) and summary (L) of mitochondrial membrane polarisation measured by MitoSpy Red incorporation (gMFI, *n* = 8). One‐way ANOVA with Tukey's correction for multiple comparisons for (C), (D) and (F). Two‐way ANOVA with Tukey's correction for multiple comparisons for (A), (B), (E), (G), (H), (J) and (L). *p < 0.05; **p < 0.01. S1P, sphingosine‐1‐phosphate. gMFI, geometric mean fluorescence intensity.

We used SCENITH to examine the metabolic phenotype of Th1‐like Tregs and the effect of FTY720 in their dependence on specific metabolic pathways. SCENITH uses protein translation measured as puromycin incorporation as a readout for ATP production [41] and determines the dependence of cells on mitochondrial respiration or glycolysis based on the use of inhibitors that specifically suppress those pathways. Th1‐like Tregs did not display differences in their energetic status measured as puromycin incorporation as compared to Tregs, and FTY720 did not have any effect either (Figure 4A). However, Th1‐like Tregs significantly decreased their mitochondrial dependence, suggesting that there is a decrease in the proportion of ATP production that is dependent on oxidative phosphorylation (OXPHOS). FTY720 significantly restored this impairment to levels comparable to those of control Tregs (Figure 4B). The decrease in mitochondrial dependence was confirmed using Seahorse assays (Figure 4C). Basal OCR was significantly decreased in in vitro‐generated Th1‐like Tregs as compared to control Tregs and FTY720 treatment of Th1‐like Tregs restored it. Moreover, after inhibition of ATP synthase with oligomycin to determine the portion of basal respiration that is used to generate ATP (Figure 4D), a decrease in the ATP produced by the mitochondria was observed in Th1‐like Tregs, which was further restored to control Treg levels by FTY720 (Figure 4D). Interestingly, no differences in glucose dependence (proportion of ATP production that is dependent on glucose oxidation) were observed in Th1‐like Tregs (Figure 4E) compared to control Tregs, and accordingly, basal extracellular acidification rate (ECAR) was similar in control and Th1‐like Tregs, with FTY720 treatment not having a significant effect (Figure 4F). However, Th1‐like Tregs displayed a significant increase in their glycolytic capacity (maximum capacity to sustain ATP production when mitochondrial respiration is inhibited) relative to control Tregs and this was inhibited by FTY720 treatment (Figure 4G). No changes in the overall capacity to use fatty acids and amino acids as fuels for ATP production when glucose oxidation is inhibited were observed in Th1‐like Tregs.

To investigate the impairment in mitochondrial respiration in Th1‐like Tregs in more detail, we measured mitochondrial mass (Figure 4I,J) and mitochondrial membrane potential (Figure 4K,L) [42]. We observed no significant changes in mitochondrial mass in Th1‐like Tregs compared to control Tregs (Figure 4I), but FTY720 increased mitochondrial mass in both Tregs and Th1‐like Tregs (Figure 4J). Unexpectedly, mitochondrial membrane potential was not impaired in Th1‐like Tregs compared to control Tregs, but was slightly decreased by FTY720 treatment.

These results suggest that Th1‐like Tregs undergo mitochondrial uncoupling characterised by a dissociation between mitochondria respiration‐dependent ATP production and mitochondrial membrane potential. S1P signalling inhibition by FTY720 rebalances mitochondrial metabolism of Th1‐like Tregs by increasing mitochondrial dependence and decreasing mitochondrial polarisation.

### 1.4 FTY720 Rebalances Th1‐Like Treg Mitochondrial Metabolism In Vivo

**FIGURE 5:**
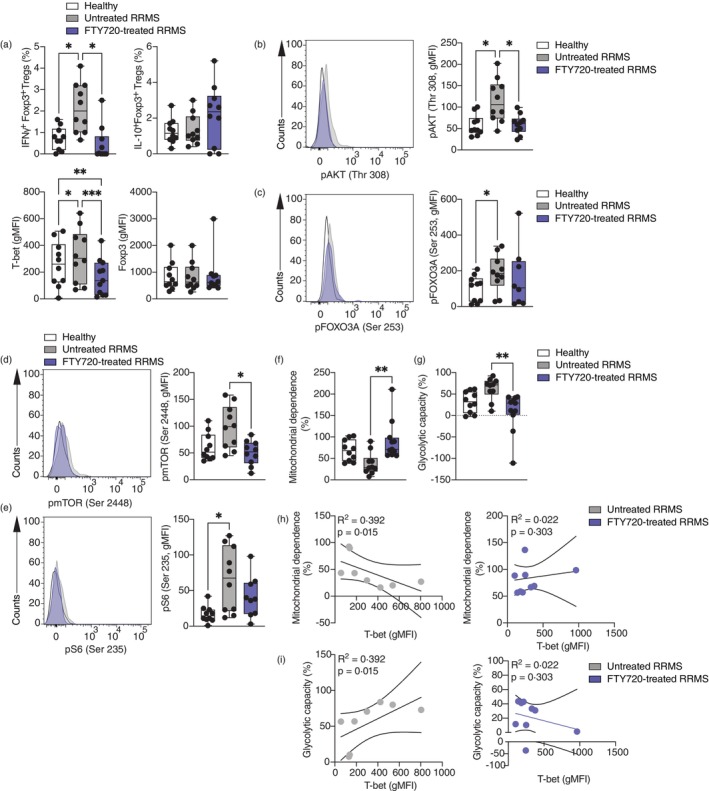
In vivo FTY720 treatment restores mitochondrial imbalance in Tregs from RRMS patients. Sorted Tregs from healthy individuals (white) and patients with RRMS either untreated (grey) or treated with FTY720 (blue) were stimulated with PMA and ionomycin in the presence of GolgiStop for 4 h (A) or left unstimulated (B–G). (A) Summary of percentage of IFNγ and IL‐10 production and T‐bet and FOXP3 expression (gMFI, *n* = 10–11). (B) Representative histogram (left) and summary (right) of phosphorylation of AKT measured at Thr 308 (*n* = 10). (C) Representative histogram (left) and summary (right) of phosphorylation of FOXO3A (*n* = 8–10). (D) Representative histogram (left) and summary (right) of mTOR phosphorylation (*n* = 10). (E) Representative histogram (left) and summary (right) of S6 phosphorylation (*n* = 9–10). (F) Summary of percentage of mitochondrial dependence (*n* = 10–11). (G) Summary of percentage of glycolytic capacity (*n* = 10–11). (H) Correlation of mitochondrial dependence (percentage) with T‐bet expression (gMFI) in untreated (left) and FTY720‐treated (right) RRMS patients. (I) Correlation of glycolytic capacity (percentage) with T‐bet expression (gMFI) in untreated (left) and FTY720‐treated (right) RRMS patients. One‐way ANOVA with Tukey's correction for multiple comparisons for (A), (C), (D), (E), (F) and (G). Spearman correlation for (H) and (I). *p < 0.05; **p < 0.01; ***p < 0.005. gMFI, geometric mean fluorescence intensity; RRMS, relapsing–remitting multiple sclerosis.

Patients with RRMS have an increased frequency of Th1‐like Tregs in vivo [3]. Therefore, in order to determine whether FTY720 also led to a modification of mitochondrial function in vivo, we carried out a cross‐sectional study examining Tregs from healthy individuals and patients with RRMS either untreated or treated with FTY720 (Table 1). In agreement with our previous data [32], untreated RRMS patients displayed increased levels of IFNγ‐producing Tregs and higher expression of T‐bet compared to Tregs isolated from healthy individuals ex vivo (Figure 5A). FTY720 treatment inhibited Th1‐like Tregs in vivo, and Tregs isolated from FTY720‐treated RRMS patients produced significantly less IFNγ and downregulated T‐bet expression, whilst no changes were observed in FOXP3 and IL‐10 protein levels (Figure 5A). Moreover, in vivo FTY720 treatment inhibited the aberrant AKT activation observed in Tregs from untreated RRMS patients compared to healthy individuals (Figure 5B), but no significant changes were observed in FOXO3A phosphorylation in Tregs from FTY720‐treated compared to untreated RRMS patients (Figure 5C), in agreement with the in vitro data (Figure 2). We went on to determine the activation status of mTORC1 (Figure 5D,E). mTOR phosphorylation showed a trend towards increased expression in untreated RRMS patients, and FTY720 treatment significantly reduced it. This was accompanied by an increased phosphorylation of S6 in untreated RRMS patients compared to healthy individuals. FTY720‐treated patient Tregs displayed a trend towards decreased pS6 as compared to untreated RRMS patients. When exploring the metabolic phenotype of Tregs from the three groups of patients, we found that FTY720‐treated RRMS patients showed a significant increase in mitochondrial dependence and a decrease in glycolytic capacity compared to untreated RRMS patients, in agreement with the data generated in vitro (Figure 5F,G). Finally, in order to determine whether the extent of metabolic alterations observed in RRMS patients correlated with the presence of Th1‐like Tregs, we tested whether mitochondrial changes, that is mitochondrial dependence and glycolytic capacity correlated with T‐bet expression as a readout for the size of the Th1‐like Treg population. Mitochondrial dependence showed a negative correlation with T‐bet expression in untreated RRMS patients, suggesting that Tregs from those untreated RRMS patients with increased Th1‐like phenotype also display lower mitochondrial dependence. This correlation was lost in patients treated with FTY720 (Figure 5H). Similarly, glycolytic capacity positively correlated with T‐bet expression only in untreated RRMS patients (Figure 5I).

**TABLE 1:** Patient characteristics.

	Fingolimod	Untreated
Total number	12	10
Age on collection (years)		
Mean (SD)	49 (8)	39 (9)
Minimum–maximum	37–58	26–57
Sex		
Male	3 (25%)	2 (20%)
Female	9 (75%)	8 (80%)
Ethnicity		
White (Caucasian)	8 (67%)	5 (50%)
White(other)	1 (8%)	2 (20%)
Asian (other)	2 (17%)	2 (20%)
Black/African/Caribbean	1 (8%)	1 (10%)
Age on diagnosis (years)		
Mean (SD)	37 (9)	38 (9)
Minimum–maximum	23–54	26–54

Abbreviation: SD, standard deviation.

These data suggest that in vivo FTY720 treatment inhibits the Th1‐like phenotype in RRMS patients by targeting the PI3K/AKT/mTORC1 pathway and rebalancing mitochondrial metabolism to reverse mitochondrial uncoupling.

## 2 Discussion

In this work we demonstrate that S1P signalling can modulate human Treg stability and function in vitro and in vivo, as its inhibition with FTY720 prevents the generation of a dysfunctional and pro‐inflammatory Th1‐like Treg phenotype by inhibiting mTORC1 signalling and reversing Th1‐like Treg mitochondrial uncoupling.

The main reported role of S1P signalling in immune cells is controlling lymphocyte migration. However, increasing evidence suggests that S1P signalling is involved in other migration‐independent cellular processes in immune cells [24]. Moreover, sphingolipid metabolism has been found to be dysregulated in many human diseases, including cancer, inflammation, atherosclerosis and asthma [43], with therapies targeting S1P signalling being effective at controlling disease in MS patients [25, 26]. Thus, in vivo S1P signalling inhibition with FTY720 treatment in RRMS patients results in increased frequencies of Tregs but a pronounced depletion of effector T cells in the periphery, with changes in Treg phenotype suggestive of increased function [32]. These data support the hypothesis that S1P signalling regulates Teff and Treg migration differently, but most importantly, that S1P signalling is involved in other cellular functions besides migration. The cell type‐specific expression of S1PR as well as their differential coupling to various G proteins likely contribute to the diverse functional outcomes of S1P signalling [36]. In this regard, our data suggest that S1PR3 and S1PR4 inhibition, but not S1PR1 blockade can mimic FTY720 effects on Treg plasticity, which provides new insights into the role of additional S1P receptors in regulating Treg plasticity, in addition to the identified role of S1PR1 in regulating Treg function in rodents [29]. Moreover, whilst there are no available data on the effects of FTY720 in modulating other forms of Treg plasticity (e.g. Th17‐like and Th2‐like Treg generation), S1PR1 deletion specifically on IL‐17‐producing cells suppresses the induction of Th17 cells and renders mice resistant to EAE induction [44]. These data suggest that FTY720 inhibits the production of other cytokines besides IFNγ, potentially by signalling via different S1P receptors.

Our data suggest that Th1‐like Treg generation requires mTORC1 activation, which is inhibited by FTY720 treatment. mTORC1 is an important nutrient sensor and metabolic regulator of glucose and mitochondrial metabolism that is activated downstream of AKT. The involvement of mTORC1 in the control of Treg phenotype and function is complex and contrasting results have been reported regarding its role in inhibiting or promoting Treg development and function. These works have demonstrated that mTORC1 is required for functional competency of thymic‐derived Tregs and have highlighted the delicate control mTORC1 activity is subjected to in T cells and Tregs [39, 45]. In our experimental setting, inhibition of mTORC1 by either FTY720 or rapamycin led to an impairment in the generation of Th1‐like Tregs and restoration of their suppressive function. In agreement with these data, overactivation of mTORC1 by Treg‐specific deletion of TSC1 has been shown to cause a decrease in Treg suppressive capacity and acquisition of an effector‐like phenotype in a colitis model [46].

Mitochondrial uncoupling, the functional dissociation between the generation of mitochondrial membrane potential and its use for ATP synthesis, can be the consequence of multiple processes including proton leak, inducible leak through uncoupler proteins (UCPs) and electron leak [47]. Whilst initially thought to be detrimental for mitochondrial function, the identification of uncoupling proteins (UCP‐1 to 5 in humans) suggests that mitochondrial uncoupling is involved in other physiological processes [47]. For example, the expression of some uncoupling proteins such as UCP‐2 have been shown to be induced by antigen stimulation of CD8^+^ T cells, suggesting that mitochondrial uncoupling is part of the physiological response to antigen [48]. In addition, polyclonal stimulation of both CD4^+^ and CD8^+^ T cells in vitro increases UCP‐2 expression [49]. Little is known about how mitochondrial uncoupling controls the function of immune cells, and what endogenous pathways control mitochondrial uncoupling in immune cells. Our results suggest that mitochondrial uncoupling is involved in the generation of dysfunctional Th1‐like Tregs and potentially favoured by the S1P‐mTORC1 signalling axis, as inhibition with FTY720 restores the mitochondrial metabolic imbalance in vitro and in vivo. Questions remain related to the specific mechanism that S1P signalling triggers to favour mitochondrial uncoupling during Th1‐like Treg reprogramming and the contribution of mTORC1 signalling.

Increasing evidence associates mitochondrial dysfunction to a number of inflammatory, autoimmune and chronic conditions [50, 51, 52, 53]. In the case of RRMS patients, our data are in agreement with works demonstrating that T cells and Tregs from treatment‐naïve patients show decreased glycolysis and OCR ex vivo [54, 55], suggesting that mitochondrial targeting in RRMS and potentially in other autoimmune conditions that present with similar Treg phenotypes [56], could be considered as a potential novel therapeutic target.

## 3 Methods

### 3.1 Patient Sample and Clinical Data Collection

Patients and healthy donors who met the eligibility criteria were recruited and provided informed consent (ethics approval obtained from the South Central – Berkshire Research Ethics Committee, REC reference number 20/SC/0308). Peripheral blood was collected from all participants and processed following a common standard operating protocol (Table 1).

### 3.2 Cell Culture Reagents and Antibodies

Cells were cultured in RPMI 1640 supplemented with 10 mM HEPES buffer sodium, 0.05 mM non‐essential amino acids, 1 mM sodium pyruvate, 2 mM L‐glutamine, 100 U/mL and 100 μg/mL penicillin/streptomycin respectively (all from GIBCO) and 5% human heat‐inactivated AB serum (Sigma). The antibodies used for stimulation were anti‐human CD3 (clone UCHT1, plate bound) and anti‐human CD28 (clone 28.2, BD Biosciences, San Jose, CA) at 1 μg/mL. Treg Inspector Beads (Miltenyi Biotec, Bergisch Gladbach, Germany) were used in suppression assays following manufacturer's recommendations. FTY720 (Sigma‐Aldrich) was used at 100 and 500 ng/mL. Rapamycin (Sigma‐Aldrich) was used at 20 and 100 nM. IL‐2 was obtained through the AIDS Research and Reference Reagent Program, Division of AIDS, National Institute of Allergy and Infectious Diseases (NIAID), National Institutes of Health (NIH) and was used at 50 U/mL. The antibodies used in this work are summarised in Table 2.

**TABLE 2:** Antibodies used in this work.

Assay	Target	Clone	Fluorochrome	Manufacturer	Catalogue #
Treg phenotyping, SCENITH	CD3		BV785	Biolegend	300471
Treg phenotyping, SCENITH	CD25		PE	Biolegend	356104
Treg phenotyping, SCENITH	CD127		FITC	BD Phamingen	560549
Treg phenotyping	T‐bet		PE‐Cy7	Invitrogen	25‐5825‐82
Treg phenotyping	Foxp3		AF700	eBioscience	56‐4776‐41
Treg phenotyping	IL‐10		APC	BD Pharmingen	554707
Treg phenotyping	IFNγ		BV605	Biolegend	505839
SCENITH	CD4		V500	BD Horizon	560769
SCENITH	CD45RO		BV421	BD Horizon	562641
SCENITH	T‐bet		PerCP‐Cy5.5	Invitrogen	25‐5825‐82
SCENITH	FoxP3		PE‐Cy7	eBioscience	45‐4776‐73
SCENITH	Puromycin		AF647	Gift from Dr. Argüello	N/A
Phosflow	FoxP3		eFluor450	eBioscience	48‐4777‐42
Phosflow	CD25		AF700	BD Pharmingen	561398
Phosflow	FoxO3a (pS253)		AF488	Bioss	bs‐3140R‐A488
Phosflow	S6 (pS235/S236)		AF647	BD Biosciences	56043
Phosflow	AKT (pS473)		PE	BD Biosciences	560378
Phosflow	mTOR (pS2448)		AF647	BD Biosciences	564242
Phosflow	AKT (pT308)		PE	BD Biosciences	558275

### 3.3 PBMC Isolation, Storage and Thawing

Peripheral blood mononuclear cells (PBMCs) were isolated by Ficoll‐Paque PLUS (Cytiva) gradient centrifugation less than 2 h after blood collection. The PBMC layer was collected, washed with PBS, resuspended at 20 million cells/mL in fetal bovine serum supplemented with 10% DMSO and stored at −150°C.

For PBMC thawing, vials were thawed in a pre‐warmed water bath at 37°C, transferred to 15 mL conical tube and 6 mL of pre‐warmed complete media was added. The tubes were subsequently centrifuged for 10 min at 300 × *g* and resuspended in warm complete media at 5 million cells/mL.

### 3.4 Flow Cytometry Staining for Regulatory T Cell Immunophenotyping

PBMCs were thawed and CD4^+^ T cells were isolated by immunomagnetic negative selection (EasySep Human CD4^+^ T Cell Enrichment Kit, StemCell Technologies); 100 000–200 000 CD4^+^ T cells per well were plated in 96‐well V‐bottom plates and rested for 1.5 h at 37°C, 5% CO_2_ in complete media. For ex vivo phenotypic characterisation, the cells were stained immediately after resting according to the protocol described hereafter, with the antibodies detailed in Table 2. For the characterisation of the cytokine profile, the cells were incubated for 4 h in 200 μL of complete media with 50 ng/mL PMA and 250 ng/mL ionomycin in the presence of GolgiStop and stained immediately after with antibodies detailed in Table 2.

All centrifugation steps in this protocol were carried out at 300 × *g* for 10 min. First, cells were stained with LIVE/DEAD Fixable Blue Dead Cell Dye (Thermo Fisher Scientific) according to the manufacturer's protocol. A Fc receptor (FcR) blocking step with FcR Blocking Reagent Human (Miltenyi Biotec) was performed with the cell surface antibody staining. The cells were subsequently fixed with the Foxp3/Transcription Factor Staining Buffer Set (Thermo Fisher Scientific) according to the manufacturer's protocol. Where relevant, an additional step for intracellular staining was added after fixation, using the FoxP3 staining buffer kit. The cells were then washed and resuspended in 250 μL of PBS.

The samples were run on a Fortessa instrument (BD Biosciences) and analysed using FlowJo v10.0 (BD Biosciences). Tregs were gated on size and granularity (lymphocyte gate), single cells, live cells, CD3^+^CD4^+^CD25^high^CD127^low^.

### 3.5 In Vitro Th1‐Like Treg Generation

Fresh PBMC were isolated and CD4^+^ T cells were enriched by immunomagnetic negative selection as described above. CD4^+^ T cells were stained with antibodies to CD4, CD25 and CD127 and Tregs (CD4^+^CD25^high^CD127^low^) and conventional T cells (Tconv, Treg‐depleted CD4^+^ T cells) were sorted on a FACS Aria II. Sorted Treg cells were plated in 96‐well U‐bottom plates (50 000 cells/well) pre‐coated with 1 μg/mL plate‐bound anti‐CD3 (clone UCHT1), and stimulated in complete media supplemented with soluble anti‐CD28 (1 μg/mL), with 50 U/mL IL‐2 and with or without 20 ng/mL IL‐12 for 4 days at 37°C, 5% CO_2_. Some wells also received FTY720 or rapamycin besides IL‐12. Cells were lysed 48 h after stimulation for gene expression analysis. After 4 days in culture, cells were restimulated with 50 ng/mL PMA and 250 ng/mL ionomycin in the presence of GolgiStop for the characterisation of the cytokine profile.

### 3.6 Metabolic Profiling Using SCENITH

SCENITH is a flow cytometry‐based assay for profiling cell energy metabolism with single cell resolution [41]. Briefly, rapid changes in protein translation upon metabolic pathway inhibition are monitored using puromycin incorporation to newly synthesised proteins as a reliable readout for protein synthesis, which is tightly coupled to ATP production and therefore can be used as a readout for the impact of metabolic pathway inhibition on the energetic status of the cells.

PBMCs were thawed and plated at 300 000–500 000 cells per well in 96‐well V‐bottom plates and rested for 1.5 h at 37°C, 5% CO_2_ in complete media. The cells were then treated for 45 min at 37°C, 5% CO_2_ with vehicle (Control, Co), 100 mM 2‐deoxy‐d‐glucose (DG, Sigma‐Aldrich), 1 μM oligomycin (O, Sigma‐Aldrich) or a combination of both drugs (DGO); 10 μg/mL puromycin was added to all conditions. Subsequently, cells were pelleted by centrifugation for 7 min at 400 × *g* and stained according to the published protocol [41, 57]. Cells were washed, resuspended in 250 μL of PBS and run on a Fortessa instrument (BD Biosciences). Data were analysed using FlowJo v10.0 (BD Biosciences). Tregs were gated based on size and granularity, singlets, live cells, CD3^+^CD4^+^CD25^high^CD127^low^.

For the analysis of the energetic status of the cells, puromycin geometric mean fluorescence intensity was analysed in each of the four conditions mentioned above (Co, DG, O, DGO). The percentage of glucose dependence was calculated using this formula: 100 × [(Co − DG)/(Co − DGO)]; and the percentage of mitochondrial dependence was calculated as 100 × [(Co − O)/(Co − DGO)]. Finally, glycolytic capacity (%) was defined as 100 − mitochondrial dependence, and fatty acid and amino acid oxidation capacity (%) was defined as 100 − glucose dependence.

### 3.7 Quantification of mRNA Expression by Real‐Time PCR

RNA was isolated using the RNeasy Micro Plus Kit (Qiagen) following the manufacturer's guidelines in Appendix D of the Qiagen RNeasy handbook. Isolated RNA was converted to complementary DNA by reverse transcription (RT) with random hexamers and Multiscribe RT (TaqMan Reverse Transcription Reagents, Thermo Fisher Scientific). The reactions were set up using the manufacturer's guidelines and run on a QS5 Studio Real‐Time PCR instrument (Thermo Fisher Scientific). Values are represented as the difference in cycle threshold (Ct) values normalised to *B2M* expression for each sample as per the following formula: Relative RNA expression = (2 − ΔCt) x 10^3^.

### 3.8 Flow Cytometry‐Based Phosphorylation Assays

For phosphorylation assays with patient samples (Figure 5), PBMCs were thawed and CD4^+^ T cells were isolated by immunomagnetic negative selection (EasySep Human CD4^+^ T Cell Enrichment Kit, StemCell Technologies); 250 000–500 000 CD4^+^ T cells per well were plated in 96‐well V‐bottom plates, rested for 2 h at 37°C, 5% CO_2_ in complete media, and fixed immediately after. For phosphorylation assays of Th1‐like Tregs, PBMC were isolated and CD4^+^ T cells were isolated fresh as above. Subsequently, 250 000–500 000 CD4^+^ T cells per well were plated in 96‐well U‐bottom plates pre‐coated with 1 μg/mL plate‐bound anti‐CD3 (clone UCHT1), and stimulated in complete media supplemented with soluble anti‐CD28 (1 μg/mL), with 50 U/mL IL‐2 and with or without 20 ng/mL IL‐12 for 18 h at 37°C, 5% CO_2_. Some wells also received FTY720 besides IL‐12. Eighteen hours later, the cells were fixed following the protocol described hereafter.

For all conditions, cells were fixed in pre‐warmed Fix Buffer I (BD Phosflow) for 20 min at 37°C, 5% CO_2_ and permeabilized with ice‐cold Perm Buffer III (BD Phosflow) overnight at −20°C. Cells were subsequently stained in PBS for 1 h at room temperature with the antibodies detailed in Table 2. Subsequently, cells were washed in PBS and resuspended in 250 μL PBS. The samples were run on a Fortessa instrument (BD Biosciences) and analysed using FlowJo v10.0 (BD Biosciences). Tregs were gated on size and granularity, singlets, live cells, CD25^high^FoxP3^+^.

### 3.9 Suppression Assays

PBMCs were thawed and stained with antibodies recognising CD3, CD4, CD25 and CD127. Tregs (CD3^+^CD4^+^CD25^high^ CD127^low^) and Tresp (CD3^+^CD4^+^CD25^−/int^CD127^+^) were sorted using a FACS Aria Fusion flow cytometer (BD Biosciences). Propidium iodide was added right before the sort to exclude dead cells.

Subsequently, Tconv were stained with CellTrace CFSE, and a suppression assay was set up in a 96‐well round‐bottom plate with five different conditions: Tconv only (0:1), co‐cultures of Tconv and Tregs at three different Treg:Tconv ratios (1:2, 1:4, 1:8); and Tconv without stimulation as a negative control for proliferation. Treg Suppression Inspector beads (Miltenyi Biotec) were added to all conditions (except Tconv without stimulation) at a 2:1 ratio to the cells. The cells were cultured in 200 μL complete media for 3.5 days and were then stained according to the protocol described for Treg immunophenotyping, with antibodies to CD4, CD25 and Ki67. The samples were run on a Fortessa instrument (BD Biosciences) and analysed using FlowJo v10.0 (BD Biosciences). Tconv were gated on size and granularity, singlets, live cells, CD4^+^ and CFSE^+^. Suppressive capacity (%) was calculated using this formula: 100 − [(Co‐culture division/Tconv only division) × 100].

### 3.10 Statistical Analysis

Data were analysed using GraphPad Prism version 10. Normal distribution of the data was tested using the Anderson‐Darling and D'Agostino and Pearson normality tests, or Shapiro–Wilk test for those datasets with a small number of replicates. Normally distributed data by at least one of the two tests was analysed by one‐ or two‐way ANOVA when comparing more than two groups of one or two independent variables, respectively. A two‐tailed *t*‐test was used to compare two groups. Data were expressed as mean ± S.E.M. Where data are presented as box and whiskers, the boxes extend from the 25th to the 75th percentile and the whiskers are drawn down to the minimum and up to the maximum values. Horizontal lines within the boxes denote the median. *p* values > 0.05 were considered statistically significant.

## Author Contributions

R.C. performed experiments, analysed the data and wrote the manuscript. N.G. and C.S. performed experiments and analysed the data. N.N., A.A.M. and A.K.M. performed experiments. R.A. provided anti‐puromycin antibody and advised on SCENITH data generation and analysis. A.S., R.N. and J.V. recruited RRMS patients and provided advice on the clinical aspects of the work. M.D.V. designed the study, analysed data, wrote the manuscript and obtained funding. All authors revised and contributed to the editing of the manuscript.

## Conflicts of Interest

The authors declare no conflicts of interest.

## Supporting information


**Figure S1.** FTY720 treatment does not affect Treg viability. Sorted Tregs from healthy individuals were stimulated with anti‐CD3, anti‐CD28 and IL‐2 in the presence or absence of IL‐12 and FTY720 for 4 days and viability was examined after 4 h of PMA and ionomycin stimulation. Representative dot plots (A) and summary (B) of percentage of live Tregs. One‐way ANOVA with Tukey’s correction for multiple comparisons.

**Figure S2.** FTY720 treatment does not alter FOXP3 expression. Sorted Tregs from healthy individuals were stimulated with anti‐CD3, anti‐CD28 and IL‐2 in the presence or absence of IL‐12 and FTY720 for 4 days and FOXP3 expression was examined after 4 h of PMA and ionomycin stimulation. Summary of *n* = 8 experiments. One‐way ANOVA with Tukey’s correction for multiple comparisons.

**Figure S3.** The effects of FTYY720 are Th1‐like Treg‐specific. Sorted Tregs were stimulated with anti‐CD3 and anti‐CD28 in the presence of IL‐2 (50 U/mL) or IL‐2 and IL‐12 (20 ng/mL, to induce Th1‐like Tregs) together or not with 500 ng/mL FTY720. (A) Cytokine expression was examined after a 4 h stimulation with PMA and ionomycin in the presence of GolgiStop after 4 days. (B) Ki67 expression was measured by flow cytometry after 2 days of stimulation. *N* = 6 donors. Two‐way ANOVA with Tukey’s correction for multiple comparisons. *p < 0.05.

**Figure S4.** S1P receptor gene expression. Gene expression of all five S1P receptors in sorted Tregs and Th1‐like Tregs from healthy individuals stimulated with anti‐CD3, anti‐CD28 and IL‐2 in the presence or absence of IL‐12 and FTY720 for 24 h, measured by real‐time PCR. *S1PR2* and *S1PR5* were not detected. Summary of *n* = 5 experiments. Two‐way ANOVA with Tukey’s correction for multiple comparisons.

**Figure S5.** S1PR3 and S1PR4 inhibitors recapitulate the inhibition of Th1‐like Treg generation mediated by FTY720. Sorted Tregs were stimulated with anti‐CD3 and anti‐CD28 in the presence of IL‐2 (50 U/mL) or IL‐2 and IL‐12 (20 ng/mL) together or not with specific inhibitors of S1PR1 (EX 26), S1PR3 (CYM 50358) or S1PR4 (TY 52156). T‐bet expression was assessed by flow cytometry after 4 days. *n* = 6 donors. Two‐way ANOVA with Tukey’s correction for multiple comparisons. *p < 0.05; **p < 0.005; ***p < 0.001.

**Figure S6.** Gating strategy for suppression analysis.

## Data Availability

The data that support the findings of this study are available from the corresponding author upon reasonable request.

## References

[1] M. Dominguez‐Villar and D. A. Hafler , “Regulatory T Cells in Autoimmune Disease,” Nature Immunology 19 (2018): 665–673. 29925983 10.1038/s41590-018-0120-4 PMC7882196

[2] A. Kitz , M. de Marcken , A. S. Gautron , M. Mitrovic , D. A. Hafler , and M. Dominguez‐Villar , “AKT Isoforms Modulate Th1‐Like Treg Generation and Function in Human Autoimmune Disease,” EMBO Reports 17 (2016): 1169–1183. 27312110 10.15252/embr.201541905 PMC4967959

[3] M. Dominguez‐Villar , C. M. Baecher‐Allan , and D. A. Hafler , “Identification of T Helper Type 1‐Like, Foxp3+ Regulatory T Cells in Human Autoimmune Disease,” Nature Medicine 17 (2011): 673–675. 10.1038/nm.2389 PMC3675886 21540856

[4] S. A. McClymont , A. L. Putnam , M. R. Lee , et al., “Plasticity of Human Regulatory T Cells in Healthy Subjects and Patients With Type 1 Diabetes,” Journal of Immunology 186 (2011): 3918–3926. 10.4049/jimmunol.1003099 PMC3091943 21368230

[5] N. Komatsu , K. Okamoto , S. Sawa , et al., “Pathogenic Conversion of Foxp3+ T Cells Into TH17 Cells in Autoimmune Arthritis,” Nature Medicine 20 (2014): 62–68. 10.1038/nm.3432 24362934

[6] K. G. MacDonald , N. A. J. Dawson , Q. Huang , J. V. Dunne , M. K. Levings , and R. Broady , “Regulatory T Cells Produce Profibrotic Cytokines in the Skin of Patients With Systemic Sclerosis,” Journal of Allergy and Clinical Immunology 135 (2015): 946–955 e949. 25678090 10.1016/j.jaci.2014.12.1932

[7] A. S. Arterbery , A. Osafo‐Addo , Y. Avitzur , et al., “Production of Proinflammatory Cytokines by Monocytes in Liver‐Transplanted Recipients With De Novo Autoimmune Hepatitis is Enhanced and Induces TH1‐Like Regulatory T Cells,” Journal of Immunology 196 (2016): 4040–4051. 10.4049/jimmunol.1502276 PMC4874532 27183637

[8] M. Filippi , A. Bar‐Or , F. Piehl , et al., “Multiple Sclerosis,” Nature Reviews. Disease Primers 4 (2018): 43. 10.1038/s41572-018-0041-4 30410033

[9] A. Huynh , M. DuPage , B. Priyadharshini , et al., “Control of PI(3) Kinase in Treg Cells Maintains Homeostasis and Lineage Stability,” Nature Immunology 16 (2015): 188–196. 25559257 10.1038/ni.3077 PMC4297515

[10] S. Shrestha , K. Yang , C. Guy , P. Vogel , G. Neale , and H. Chi , “Treg Cells Require the Phosphatase PTEN to Restrain TH1 and TFH Cell Responses,” Nature Immunology 16 (2015): 178–187. 25559258 10.1038/ni.3076 PMC4297581

[11] T. Sumida , M. R. Lincoln , C. M. Ukeje , et al., “Activated Beta‐Catenin in Foxp3(+) Regulatory T Cells Links Inflammatory Environments to Autoimmunity,” Nature Immunology 19 (2018): 1391–1402. 30374130 10.1038/s41590-018-0236-6 PMC6240373

[12] S. L. Pompura and M. Dominguez‐Villar , “The PI3K/AKT Signaling Pathway in Regulatory T‐Cell Development, Stability, and Function,” Journal of Leukocyte Biology 103 (2018): 1065–1076. 10.1002/JLB.2MIR0817-349R 29357116

[13] G. M. Delgoffe , S. R. Woo , M. E. Turnis , et al., “Stability and Function of Regulatory T Cells is Maintained by a Neuropilin‐1‐Semaphorin‐4a Axis,” Nature 501 (2013): 252–256. 23913274 10.1038/nature12428 PMC3867145

[14] L. E. Lucca , P. P. Axisa , E. R. Singer , N. M. Nolan , M. Dominguez‐Villar , and D. A. Hafler , “TIGIT Signaling Restores Suppressor Function of Th1 Tregs,” JCI Insight 4 (2019): e124427. 30728325 10.1172/jci.insight.124427 PMC6413794

[15] V. A. Gerriets , R. J. Kishton , A. G. Nichols , et al., “Metabolic Programming and PDHK1 Control CD4^+^ T Cell Subsets and Inflammation,” Journal of Clinical Investigation 125 (2015): 194–207. 25437876 10.1172/JCI76012 PMC4382238

[16] R. D. Michalek , V. A. Gerriets , S. R. Jacobs , et al., “Cutting Edge: Distinct Glycolytic and Lipid Oxidative Metabolic Programs are Essential for Effector and Regulatory CD4^+^ T Cell Subsets,” Journal of Immunology 186 (2011): 3299–3303. 10.4049/jimmunol.1003613 PMC3198034 21317389

[17] C. Procaccini , F. Carbone , D. di Silvestre , et al., “The Proteomic Landscape of Human Ex Vivo Regulatory and Conventional T Cells Reveals Specific Metabolic Requirements,” Immunity 44 (2016): 712. 10.1016/j.immuni.2016.02.022 PMC5641922 28843073

[18] A. Angelin , L. Gil‐de‐Gómez , S. Dahiya , et al., “Foxp3 Reprograms T Cell Metabolism to Function in Low‐Glucose, High‐Lactate Environments,” Cell Metabolism 25 (2017): 1282–1293 e1287. 28416194 10.1016/j.cmet.2016.12.018 PMC5462872

[19] C. S. Field , F. Baixauli , R. L. Kyle , et al., “Mitochondrial Integrity Regulated by Lipid Metabolism is a Cell‐Intrinsic Checkpoint for Treg Suppressive Function,” Cell Metabolism 31 (2020): 422–437 e425. 31883840 10.1016/j.cmet.2019.11.021 PMC7001036

[20] U. H. Beier , A. Angelin , T. Akimova , et al., “Essential Role of Mitochondrial Energy Metabolism in Foxp3(+) T‐Regulatory Cell Function and Allograft Survival,” FASEB Journal 29 (2015): 2315–2326. 25681462 10.1096/fj.14-268409 PMC4447222

[21] S. E. Weinberg , B. D. Singer , E. M. Steinert , et al., “Mitochondrial Complex III is Essential for Suppressive Function of Regulatory T Cells,” Nature 565 (2019): 495–499. 30626970 10.1038/s41586-018-0846-z PMC6345596

[22] J. G. Cyster , “Chemokines, Sphingosine‐1‐Phosphate, and Cell Migration in Secondary Lymphoid Organs,” Annual Review of Immunology 23 (2005): 127–159. 10.1146/annurev.immunol.23.021704.115628 15771568

[23] C. S. Garris , V. A. Blaho , T. Hla , and M. H. Han , “Sphingosine‐1‐Phosphate Receptor 1 Signalling in T Cells: Trafficking and Beyond,” Immunology 142 (2014): 347–353. 24597601 10.1111/imm.12272 PMC4080950

[24] H. C. Tsai and M. H. Han , “Sphingosine‐1‐Phosphate (S1P) and S1P Signaling Pathway: Therapeutic Targets in Autoimmunity and Inflammation,” Drugs 76 (2016): 1067–1079. 27318702 10.1007/s40265-016-0603-2

[25] J. A. Cohen , F. Barkhof , G. Comi , et al., “Oral Fingolimod or Intramuscular Interferon for Relapsing Multiple Sclerosis,” New England Journal of Medicine 362 (2010): 402–415. 20089954 10.1056/NEJMoa0907839

[26] L. Kappos , E. W. Radue , P. O'Connor , et al., “A Placebo‐Controlled Trial of Oral Fingolimod in Relapsing Multiple Sclerosis,” New England Journal of Medicine 362 (2010): 387–401. 20089952 10.1056/NEJMoa0909494

[27] K. Chiba and K. Adachi , “Discovery of Fingolimod, the Sphingosine 1‐Phosphate Receptor Modulator and its Application for the Therapy of Multiple Sclerosis,” Future Medicinal Chemistry 4 (2012): 771–781. 22530640 10.4155/fmc.12.25

[28] J. J. Liao , M. C. Huang , and E. J. Goetzl , “Cutting Edge: Alternative Signaling of Th17 Cell Development by Sphingosine 1‐Phosphate,” Journal of Immunology 178 (2007): 5425–5428. 10.4049/jimmunol.178.9.5425 17442922

[29] G. Liu , S. Burns , G. Huang , et al., “The Receptor S1P1 Overrides Regulatory T Cell‐Mediated Immune Suppression Through Akt‐mTOR,” Nature Immunology 10 (2009): 769–777. 19483717 10.1038/ni.1743 PMC2732340

[30] M. Aoki , H. Aoki , R. Ramanathan , N. C. Hait , and K. Takabe , “Sphingosine‐1‐Phosphate Signaling in Immune Cells and Inflammation: Roles and Therapeutic Potential,” Mediators of Inflammation 2016 (2016): 8606878. 26966342 10.1155/2016/8606878 PMC4761394

[31] A. Weigert , C. Olesch , and B. Brune , “Sphingosine‐1‐Phosphate and Macrophage Biology—How the Sphinx Tames the Big Eater,” Frontiers in Immunology 10 (2019): 1706. 31379883 10.3389/fimmu.2019.01706 PMC6658986

[32] M. Dominguez‐Villar , K. Raddassi , A. C. Danielsen , J. Guarnaccia , and D. A. Hafler , “Fingolimod Modulates T Cell Phenotype and Regulatory T Cell Plasticity In Vivo,” Journal of Autoimmunity 96 (2019): 40–49. 30122421 10.1016/j.jaut.2018.08.002 PMC7882197

[33] A. S. Gautron , M. Dominguez‐Villar , M. de Marcken , and D. A. Hafler , “Enhanced Suppressor Function of TIM‐3+ FoxP3+ Regulatory T Cells,” European Journal of Immunology 44 (2014): 2703–2711. 24838857 10.1002/eji.201344392 PMC4165702

[34] E. Sawicka , G. Dubois , G. Jarai , et al., “The Sphingosine 1‐Phosphate Receptor Agonist FTY720 Differentially Affects the Sequestration of CD4^+^/CD25^+^ T‐Regulatory Cells and Enhances Their Functional Activity,” Journal of Immunology 175 (2005): 7973–7980. 10.4049/jimmunol.175.12.7973 16339533

[35] A. Kitz , M. de Marcken , A. S. Gautron , M. Mitrovic , D. A. Hafler , and M. Dominguez‐Villar , “AKT Isoforms Modulate Th1‐Like Treg Generation and Function in Human Autoimmune Disease,” EMBO Reports 20 (2019): e48624. 31379130 10.15252/embr.201948624 PMC6680151

[36] V. Brinkmann , “Sphingosine 1‐Phosphate Receptors in Health and Disease: Mechanistic Insights From Gene Deletion Studies and Reverse Pharmacology,” Pharmacology & Therapeutics 115 (2007): 84–105. 17561264 10.1016/j.pharmthera.2007.04.006

[37] B. D. Manning and A. Toker , “AKT/PKB Signaling: Navigating the Network,” Cell 169 (2017): 381–405. 28431241 10.1016/j.cell.2017.04.001 PMC5546324

[38] W. Ouyang , O. Beckett , Q. Ma , J. H. Paik , R. A. DePinho , and M. O. Li , “Foxo Proteins Cooperatively Control the Differentiation of Foxp3+ Regulatory T Cells,” Nature Immunology 11 (2010): 618–627. 20467422 10.1038/ni.1884

[39] H. Zeng and H. Chi , “mTOR Signaling in the Differentiation and Function of Regulatory and Effector T Cells,” Current Opinion in Immunology 46 (2017): 103–111. 28535458 10.1016/j.coi.2017.04.005 PMC5554750

[40] H. Huang , L. Long , P. Zhou , N. M. Chapman , and H. Chi , “mTOR Signaling at the Crossroads of Environmental Signals and T‐Cell Fate Decisions,” Immunological Reviews 295 (2020): 15–38. 32212344 10.1111/imr.12845 PMC8101438

[41] R. J. Arguello , A. J. Combes , R. Char , et al., “SCENITH: A Flow Cytometry‐Based Method to Functionally Profile Energy Metabolism With Single‐Cell Resolution,” Cell Metabolism 32 (2020): 1063–1075 e1067. 33264598 10.1016/j.cmet.2020.11.007 PMC8407169

[42] L. B. Monteiro , G. G. Davanzo , C. F. de Aguiar , and P. M. M. Moraes‐Vieira , “Using Flow Cytometry for Mitochondrial Assays,” MethodsX 7 (2020): 100938. 32551241 10.1016/j.mex.2020.100938 PMC7289760

[43] K. Takabe , S. W. Paugh , S. Milstien , and S. Spiegel , ““Inside‐Out” Signaling of Sphingosine‐1‐Phosphate: Therapeutic Targets,” Pharmacological Reviews 60 (2008): 181–195. 18552276 10.1124/pr.107.07113 PMC2695666

[44] A. Eken , R. Duhen , A. K. Singh , et al., “S1P(1) Deletion Differentially Affects TH17 and Regulatory T Cells,” Scientific Reports 7 (2017): 12905. 29018225 10.1038/s41598-017-13376-2 PMC5635040

[45] H. Zeng and H. Chi , “Metabolic Control of Regulatory T Cell Development and Function,” Trends in Immunology 36 (2015): 3–12. 25248463 10.1016/j.it.2014.08.003 PMC4280284

[46] Y. Park , H. S. Jin , J. Lopez , et al., “TSC1 Regulates the Balance Between Effector and Regulatory T Cells,” Journal of Clinical Investigation 123 (2013): 5165–5178. 24270422 10.1172/JCI69751 PMC3859395

[47] S. Demine , P. Renard , and T. Arnould , “Mitochondrial Uncoupling: A Key Controller of Biological Processes in Physiology and Diseases,” Cells 8 (2019): 795. 31366145 10.3390/cells8080795 PMC6721602

[48] L. Chaudhuri , R. K. Srivastava , F. Kos , and P. A. Shrikant , “Uncoupling Protein 2 Regulates Metabolic Reprogramming and Fate of Antigen‐Stimulated CD8+ T Cells,” Cancer Immunology, Immunotherapy 65 (2016): 869–874. 27271549 10.1007/s00262-016-1851-4 PMC4919150

[49] A. Rupprecht , A. U. Bräuer , A. Smorodchenko , et al., “Quantification of Uncoupling Protein 2 Reveals Its Main Expression in Immune Cells and Selective Up‐Regulation During T‐Cell Proliferation,” PLoS One 7 (2012): e41406. 22870219 10.1371/journal.pone.0041406 PMC3411681

[50] M. J. Barrera , S. Aguilera , I. Castro , et al., “Dysfunctional Mitochondria as Critical Players in the Inflammation of Autoimmune Diseases: Potential Role in Sjogren's Syndrome,” Autoimmunity Reviews 20 (2021): 102867. 34118452 10.1016/j.autrev.2021.102867

[51] A. Diaz‐Vegas , P. Sanchez‐Aguilera , J. R. Krycer , et al., “Is Mitochondrial Dysfunction a Common Root of Noncommunicable Chronic Diseases?,” Endocrine Reviews 41 (2020): bnaa005. 32179913 10.1210/endrev/bnaa005 PMC7255501

[52] I. P. Barcelos , R. M. Troxell , and J. S. Graves , “Mitochondrial Dysfunction and Multiple Sclerosis,” Biology (Basel) 8 (2019): 37. 31083577 10.3390/biology8020037 PMC6627385

[53] S. H. Kwak , K. S. Park , K. U. Lee , and H. K. Lee , “Mitochondrial Metabolism and Diabetes,” Journal of Diabetes Investigation 1 (2010): 161–169. 24843427 10.1111/j.2040-1124.2010.00047.x PMC4020716

[54] C. La Rocca , F. Carbone , V. De Rosa , et al., “Immunometabolic Profiling of T Cells From Patients With Relapsing‐Remitting Multiple Sclerosis Reveals an Impairment in Glycolysis and Mitochondrial Respiration,” Metabolism 77 (2017): 39–46. 29132538 10.1016/j.metabol.2017.08.011 PMC5800394

[55] A. Duscha , B. Gisevius , S. Hirschberg , et al., “Propionic Acid Shapes the Multiple Sclerosis Disease Course by an Immunomodulatory Mechanism,” Cell 180 (2020): 1067–1080 e1016. 32160527 10.1016/j.cell.2020.02.035

[56] A. Kitz and M. Dominguez‐Villar , “Molecular Mechanisms Underlying Th1‐Like Treg Generation and Function,” Cellular and Molecular Life Sciences 74 (2017): 4059–4075. 28624966 10.1007/s00018-017-2569-y PMC7079789

[57] A. K. Maher , K. L. Burnham , E. M. Jones , et al., “Transcriptional Reprogramming From Innate Immune Functions to a Pro‐Thrombotic Signature by Monocytes in COVID‐19,” Nature Communications 13 (2022): 7947. 10.1038/s41467-022-35638-y PMC9791976 36572683

