# Supplementary figures and images for: Sphingosine‐1‐Phosphate Signalling Inhibition Suppresses Th1‐Like Treg Generation by Reversing Mitochondrial Uncoupling
Source: Immunology. 2024 Oct 24;174(1):153–66. doi: 10.1111/imm.13870 (PMC11652410; doi:10.1111/imm.13870)

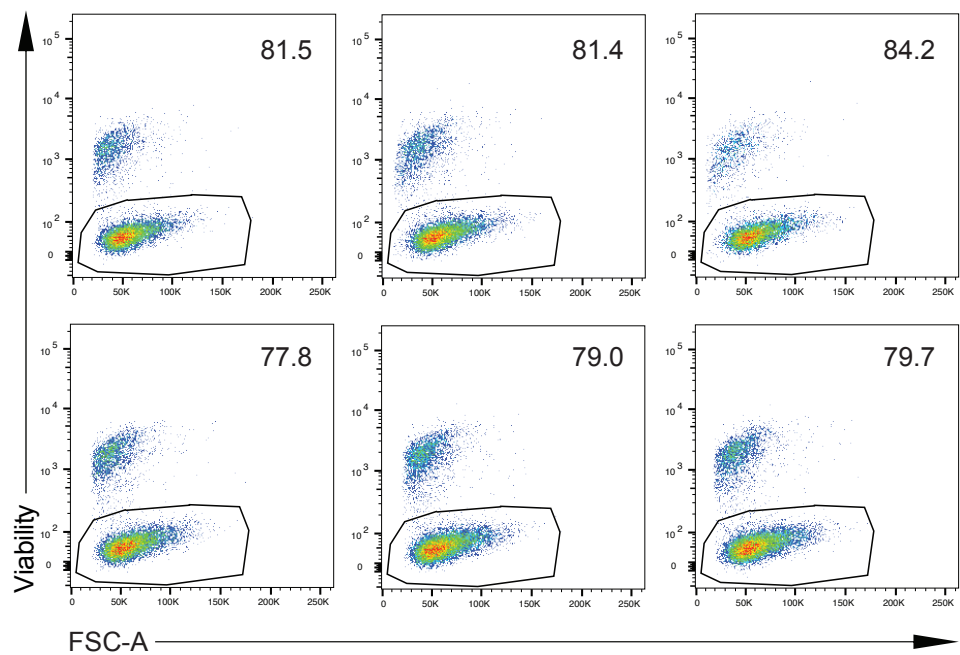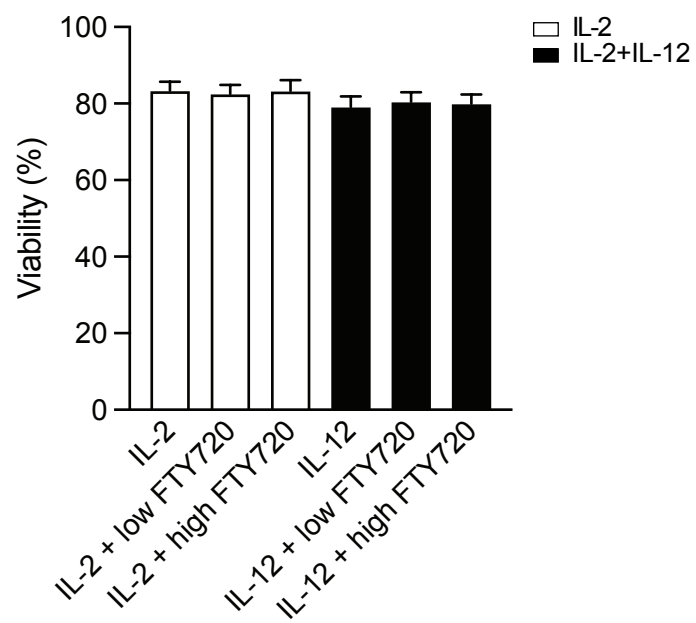

Supplement: Supplementary file 1 — Figure S1. FTY720 treatment does not affect Treg viability. Sorted Tregs from healthy individuals were stimulated with anti‐CD3, anti‐CD28 and IL‐2 in the presence or absence of IL‐12 and FTY720 for 4 days and viability was examined after 4 h of PMA and ionomycin stimulation. Representative dot plots (A) and summary (B) of percentage of live Tregs. One‐way ANOVA with Tukey’s correction for multiple comparisons. [file IMM-174-153-s002.pdf]

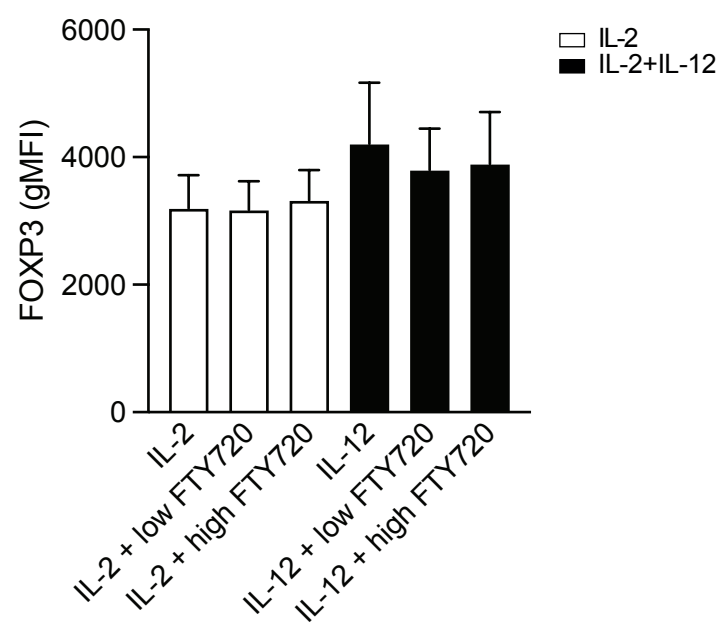

Supplement: Supplementary file 2 — Figure S2. FTY720 treatment does not alter FOXP3 expression. Sorted Tregs from healthy individuals were stimulated with anti‐CD3, anti‐CD28 and IL‐2 in the presence or absence of IL‐12 and FTY720 for 4 days and FOXP3 expression was examined after 4 h of PMA and ionomycin stimulation. Summary of n = 8 experiments. One‐way ANOVA with Tukey’s correction for multiple comparisons. [file IMM-174-153-s006.pdf]

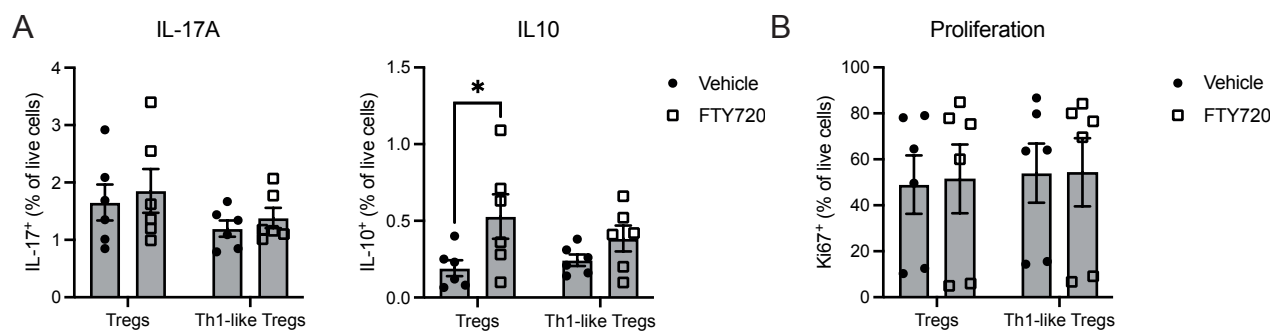

Supplement: Supplementary file 3 — Figure S3. The effects of FTYY720 are Th1‐like Treg‐specific. Sorted Tregs were stimulated with anti‐CD3 and anti‐CD28 in the presence of IL‐2 (50 U/mL) or IL‐2 and IL‐12 (20 ng/mL, to induce Th1‐like Tregs) together or not with 500 ng/mL FTY720. (A) Cytokine expression was examined after a 4 h stimulation with PMA and ionomycin in the presence of GolgiStop after 4 days. (B) Ki67 expression was measured by flow cytometry after 2 days of stimulation. N = 6 donors. Two‐way ANOVA with Tukey’s correction for multiple comparisons. *p < 0.05. [file IMM-174-153-s001.pdf]

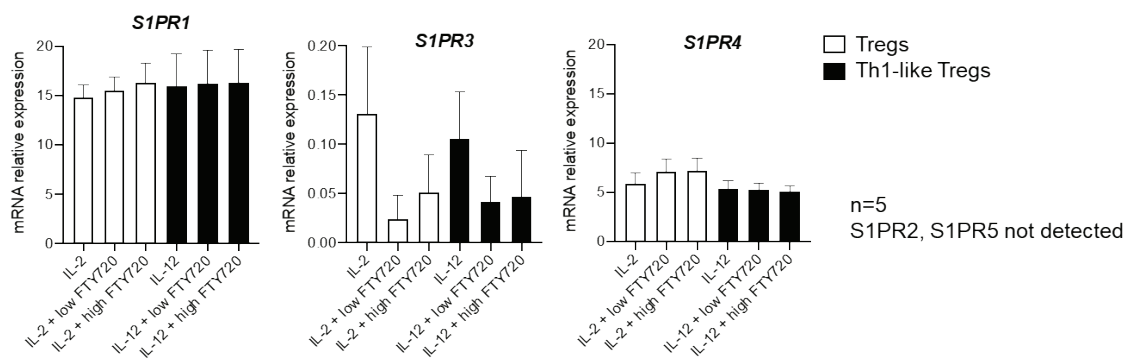

Supplement: Supplementary file 4 — Figure S4. S1P receptor gene expression. Gene expression of all five S1P receptors in sorted Tregs and Th1‐like Tregs from healthy individuals stimulated with anti‐CD3, anti‐CD28 and IL‐2 in the presence or absence of IL‐12 and FTY720 for 24 h, measured by real‐time PCR. S1PR2 and S1PR5 were not detected. Summary of n = 5 experiments. Two‐way ANOVA with Tukey’s correction for multiple comparisons. [file IMM-174-153-s005.pdf]

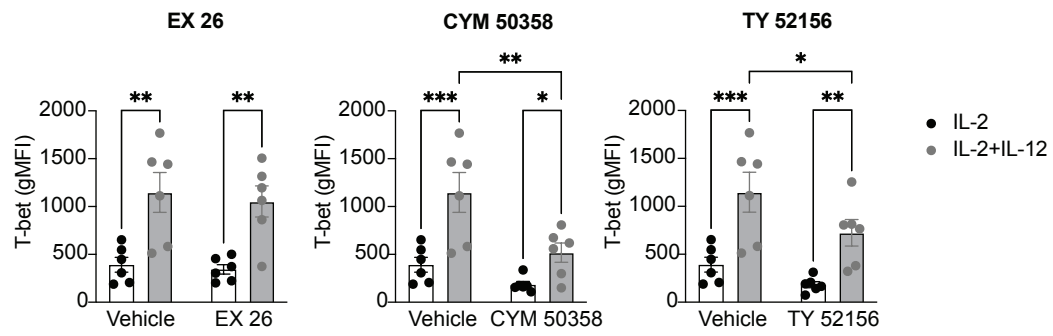

Supplement: Supplementary file 5 — Figure S5. S1PR3 and S1PR4 inhibitors recapitulate the inhibition of Th1‐like Treg generation mediated by FTY720. Sorted Tregs were stimulated with anti‐CD3 and anti‐CD28 in the presence of IL‐2 (50 U/mL) or IL‐2 and IL‐12 (20 ng/mL) together or not with specific inhibitors of S1PR1 (EX 26), S1PR3 (CYM 50358) or S1PR4 (TY 52156). T‐bet expression was assessed by flow cytometry after 4 days. n = 6 donors. Two‐way ANOVA with Tukey’s correction for multiple comparisons. *p < 0.05; **p < 0.005; ***p < 0.001. [file IMM-174-153-s004.pdf]

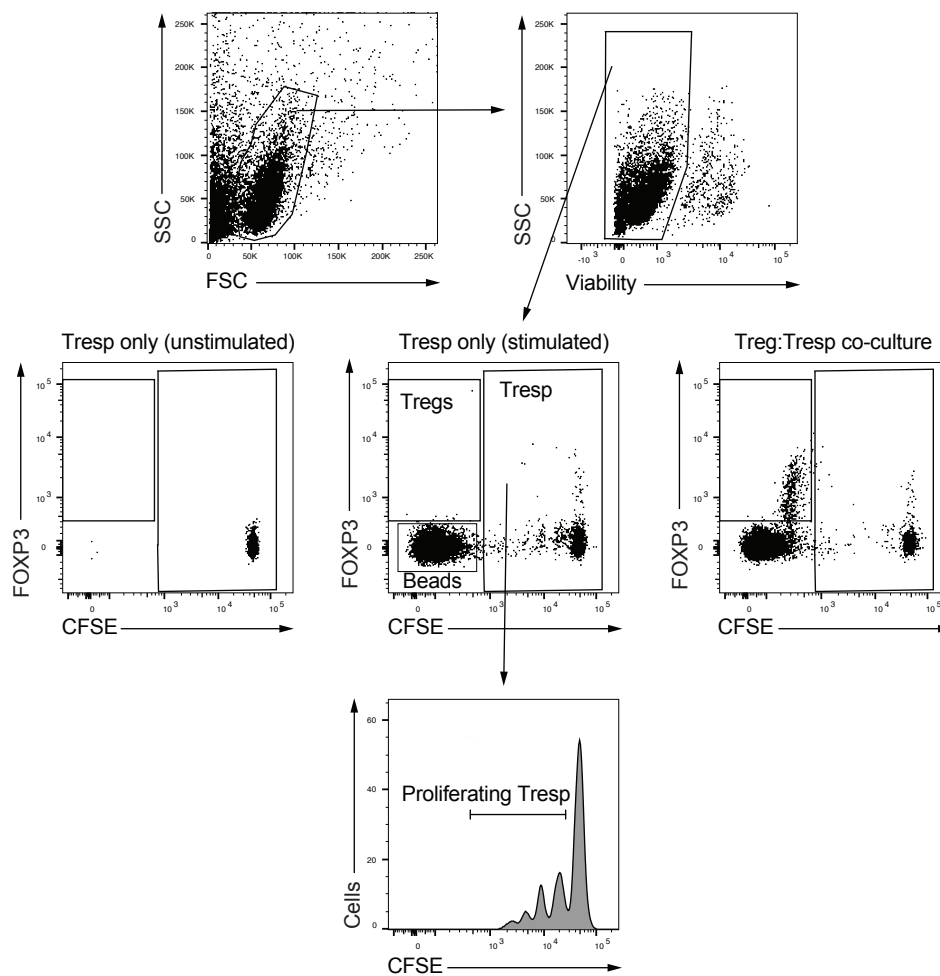

Supplement: Supplementary file 6 — Figure S6. Gating strategy for suppression analysis. [file IMM-174-153-s003.pdf]
